# Characteristics and context of fentanyl test strip use among syringe service clients in southern Wisconsin

**DOI:** 10.1186/s12954-022-00720-7

**Published:** 2022-12-15

**Authors:** Alyssa Shell Tilhou, Jen Birstler, Amelia Baltes, Elizabeth Salisbury-Afshar, Julia Malicki, Guanhua Chen, Randall Brown

**Affiliations:** 1 Department of Family Medicine, Boston University/Boston Medical Center, 771 Albany St., Dowling 5 South, Rm 5507A, Boston, MA 02118 USA; 2 Department of Biostatistics and Medical Informatics, University of Wisconsin School of Medicine and Public Health, Madison, WI USA; 3 Department of Family Medicine and Community Health, University of Wisconsin School of Medicine and Public Health, Madison, WI USA

**Keywords:** Fentanyl test strip (FTS), Opioid, Harm reduction, Drug testing, Overdose

## Abstract

**Background:**

Fentanyl adulteration of illicit drugs is a major driver of opioid-involved overdose in the USA. Fentanyl test strips are increasingly used by people who use drugs to check for fentanyl. However, little is known about factors that influence test strip use in this population.

**Methods:**

In this mixed-methods study employing semi-structured open-ended interviews (*n* = 29) and a structured survey (*n* = 341), we examined characteristics associated with test strip use, characteristics of test strip use, and situational, logistical and psychosocial factors influencing test strip use. Respondents were recruited from a syringe service program in southern Wisconsin. Bivariate tests of association and multivariable logistic regression examined the relationship between respondent characteristics and test strip use. Summary statistics were used to describe how situational, logistical and psychosocial factors impact test strip use.

**Results:**

Most respondents were male (59.6%), non-Hispanic white (77.4%), young (mean 35.7 years), reported heroin as their primary drug (70.7%), injection as their primary route (87.9%), and use ≥ 3 times daily (78.6%). In multivariable models, site, race and ethnicity, drug of choice, and seeking fentanyl were associated with test strip use. Among test strip users, 36.5% use them most of the time or more and 80.6% get positive results half the time or more. Among individuals reporting heroin, fentanyl, methamphetamine, or cocaine or crack cocaine at least once per month, 99.1%, 56.8%, 42.2%, and 55.7% reported testing these drugs, respectively. Test strip use is supported by information from suppliers, regular transportation, diverse distribution locations, recommendations from harm reduction staff, and having a safe or private place to use.

**Conclusions:**

We found that individuals who use fentanyl test strips are more often non-Hispanic white, use heroin, and seek drugs with fentanyl relative to individuals without test strip use. Findings confirm high fentanyl penetration in the Wisconsin drug supply. Low rates of stimulant testing suggest inadequate awareness of fentanyl penetration. Findings support outreach to key populations, increased diversity of distributing locations, efforts to correct misperceptions about drug wasting, emphasis on pre-consumption testing, and the importance of adjunct behaviors to prevent overdose given high rates of intentional fentanyl use.

## Background

Since 2000, opioid overdose deaths have surged from 6.2 per 100,000 persons [1] to 21.6 per 100,000 persons in 2019 [2]. This astounding rise has been primarily driven by adulteration of the illicit drug supply with potent synthetic opioids, namely fentanyl and its analogs [3, 4]. Treatment with medications for opioid use disorder (OUD) reduces opioid overdose mortality [5, 6]. Yet, less than 30% of the 2 million people living with OUD receive treatment, reflecting diverse barriers to treatment services [7, 8]. Consequently, affordable alternative approaches are critically needed to reduce opioid overdose.

Fentanyl test strips (FTS) are a harm reduction tool available to people who use drugs (PWUD). Originally an immunoassay for urine drug testing [9], FTS can detect fentanyl and some analogs in drug solution [10]. Harm reduction organizations have been distributing FTS to PWUD for the purposes of drug checking [9, 11–15]. Literature on feasibility and impact of FTS suggests that FTS may allow PWUD to reduce opioid overdose risk [9, 12]. For example, FTS use is associated with using less, injecting more slowly, using a test dose, and not using alone [9, 11, 12, 16]. FTS are also affordable and easily distributed where legal [17, 18].

Despite promising findings, challenges exist to FTS expansion. For example, stigma can discourage individuals from seeking services where they might receive or learn about FTS [19]. Criminalization and fear of prosecution also limits access to harm reduction services [20]; paraphernalia laws make FTS possession a felony in some states [17, 18]. Social determinants create additional barriers since transportation and safe housing impact the ability to access and utilize FTS [21–26]. Generally, few locations offer FTS limiting access to this tool [25, 27].

Thus far, little research explores individual level and contextual factors impacting FTS use. Knowing more about who uses FTS and associated factors can help harm reduction organizations optimize their outreach and education around FTS. This paper addresses this gap by describing the characteristics of people who use FTS, the characteristics of FTS use, and the situational, logistical and psychosocial factors that promote FTS use in southern Wisconsin.

## Methods

### Design

Data for this study come from Screening for Adulterants like Fentanyl and Risks of Fentanyl Test Strip Use (SAFeR), a sequential exploratory mixed-methods study on drug use behaviors among people who do and do not use FTS to test drugs for fentanyl. Phase 1 involved thematic analysis of interview data from semi-structured, open-ended interviews. Phase 2 incorporated content and language from Phase 1 into a survey focused on FTS use and related behaviors for distribution in a separate, larger sample. The study protocol was approved by the Institutional Review Board at the University of Wisconsin School of Medicine and Public Health (UWSMPH). SAFeR was funded by an internal grant through the Department of Family Medicine and Community Health, UWSMPH. This paper presents findings from Phase 2 survey data.

### Respondents and recruitment

Respondents were recruited through a multi-state HIV prevention and treatment organization, which houses a syringe service program (SSP) that distributes supplies including FTS. At the time of study implementation, FTS were only available through this SSP for clients. Recruitment occurred at four SSP sites in Beloit, Kenosha, Madison, and Milwaukee through flyers and verbal advertisement. Each site offered comparable services though differed in size with the urban sites (Madison and Milwaukee) larger than the rural sites (Beloit and Kenosha) in terms of staffing, facilities and client population. Inclusion criteria required being ≥ 18 years, an existing SSP client (to avoid incentivizing drug use disclosure), at least 3 cumulative months of weekly nonprescribed substance use (excluding cannabis) and weekly use over the past 30 days, ability to speak and read in English, and, in Phase 2, ability to navigate a tablet-based survey. Exclusion criteria included previous participation and impaired cognition or safety concerns due to intoxication. In Phase 1, respondents provided verbal informed consent prior to interview. They received $30 at completion. In Phase 2, respondents provided digital consent within the survey. They received $15 at completion or $5 after failing inclusion/exclusion criteria.

### Protocol

#### Phase 1 data collection

Phase 1 aimed to identify topics and concerns among PWUD that might not be apparent from the published literature on FTS, and also to learn the language used by PWUD when discussing FTS. Specifically, Phase 1 consisted of in-depth interviews about behaviors impacting overdose risk, perceptions about fentanyl and FTS, factors influencing FTS use, and FTS-related communication among peers. The interview employed two types of questions: (1) non-directive, open-ended questions encouraging respondents to discuss concepts with minimal guidance from the researcher [28], and (2) directive open-ended questions aiming to gain depth of knowledge in several prespecified domains using free-listing [29]. Responses to non-directive questions provided insight into unexpected items for incorporation into the survey. For example, this approach identified fear of wasting drugs a common concern among respondents. Responses to directive free-listing items were tabulated across interviews to identify the most frequently mentioned items and to identify the point of saturation [29]. Interviews were conducted remotely via Cisco WebEx Web Conferencing with respondents on-site at their respective SSP via telephone. Five members of the study team led interviews, which were later transcribed by a professional transcription service. Interviews (*n* = 29) were conducted August—December 2020.

#### Phase 2 survey development and data collection

Content and language elicited in Phase 1 informed Phase 2 survey scope and content. Survey questions directly incorporated terminology and phrasing used by Phase 1 respondents. Survey questions prioritized topics (1) mentioned frequently, (2) relevant for overdose safety, and (3) perceived as actionable by the study team and SSP staff. Survey questions also asked respondents about situational, logistical and psychosocial factors influencing FTS use that were identified by Phase 1 respondents. These questions aimed to assess representativeness of these factors in a larger sample.

Data extraction from Phase 1 produced a 57-item questionnaire. Survey questions assessed demographic characteristics, drug use characteristics, FTS use characteristics, drug use behaviors, peer influences, perceptions of fentanyl and overdose risk, substance use treatment engagement, and factors impacting FTS use. Two items addressed misperceptions that emerged in Phase 1: needing to waste drugs to use FTS, and using FTS to look for fentanyl analogs. To prevent miseducating respondents, survey completion (and compensation) required that respondents confirm receipt of embedded educational material explaining that (1) using FTS does not require wasting drugs and (2) that FTS cannot reliably test for fentanyl analogs.

Surveys were self-administered without assistance from research and SSP staff on a tablet computer. Upon completion, survey data were automatically stored and protected from viewing or editing within the tablet. Data were subsequently transmitted and stored using Research Electronic Data Capture (REDCap) electronic data capture tools hosted at the UWSMPH Department of Family Medicine and Community Health. REDCap is a secure, web-based software platform designed to support data capture for research studies [30, 31]. In total, 341 surveys were collected March–September 2021.

#### Community engagement

Several external groups reviewed study materials. SSP staff and leadership provided feedback on interview and survey questions, study protocol, and recruitment strategy with respect to feasibility, comprehensibility, and importance. In addition, SAFeR TSU contracted a community-based research consultation group [Community Advisors on Research Design and Strategies (CARDS)] to review the Phase 2 survey and bring community voices to the process [32]. Importantly, CARDS includes a high proportion of members from communities historically marginalized and underrepresented in academic settings due to race or ethnicity, socioeconomic status, or educational attainment. While we did not ask members to confirm personal history of substance use, many members reported exposure through their community networks. Finally, the study team received itemized feedback on survey question structure and wording from the University of Wisconsin Survey Center.

### Measures

Sociodemographic characteristics included age, gender (man, woman, nonbinary, trans, and other), race and ethnicity (American Indian or Alaskan Native, Asian, Black or African American, Hispanic or Latinx, Native Hawaiian or Other Pacific Islander, White, and other), education, employment, and self-reported county of residence. Urban county status was determined if at least half of the residents lived in census tracts with secondary Rural–Urban Commuting Codes of 1.0, 1.1, 2.0, 2.1, 3.0, 4.1, 5.1, 7.1, 8.1, and 10.1 [33].

Drug use characteristics included age at first drug use, drug of choice (herein, DOC-drug name), primary route of use, drug use frequency, polysubstance use, lifetime overdose count and recent treatments to stop or reduce drug use. Polysubstance use was defined as using one of the following substances at least monthly in addition to the DOC: heroin, fentanyl, opioid analgesics, synthetics, prescription anxiety medications, cocaine, crack cocaine, methamphetamine, prescription amphetamines or other.

### Analysis

Demographic characteristics were summarized for the sample. Associations between FTS use with demographic and drug use characteristics were determined using chi-square tests for nominal categorical variables and Mann–Whitney-Wilcoxon tests for continuous variables and categorical variables with natural ordering. Multivariable logistic regression examined associations between demographic characteristics and FTS use in adjusted models. Characteristics of FTS use were summarized for respondents who reported FTS use.

Analysis of situational and logistical factors influencing FTS use was limited to respondents who reported FTS use. Analysis of psychosocial factors influencing FTS use was assessed for the full sample. Likert responses were mapped from 0 to 4 and described by the mean and standard deviation. Associations of each factor with demographic and drug use characteristics (situational and logistical factors: 13 characteristics; psychosocial factors: 14 characteristics) were determined using Kruskal–Wallis tests for nominal variables and Jonckheere–Terpstra tests for ordinal variables (age, number of overdoses, years of drug use). Pairwise testing was conducted for significant results (*p* < 0.05) in which one of the variables consisted of three or more nominal categories. Mann–Whitney tests were used when the other variable was continuous or had a natural ordering, and chi-square tests were used when the other variable was categorical and nominal. No *p* value corrections for inflated type 1 error were used. All statistical analysis was conducted in R version 4.1.2 (2021-11-01).

## Results

### Sample characteristics

Recruitment achieved even distribution across all sites (Table 1). Of 341 respondents, 274 reported using FTS or using drugs someone else tested with FTS. The average age was 35.7 years. Most respondents identified as men (*n* = 202, 59.6%) and non-Hispanic white (*n* = 263, 77.4%). Among respondents, 7.1% (*n* = 24) were non-Hispanic Black or African American, 6.2% (*n* = 21) were Hispanic, 2.9% (*n* = 10) were non-Hispanic American Indian, 1.8% (*n* = 6) reported other, and 4.7% (*n* = 16) reported multiple categories. Only 3.2% reported a 4-year college degree or more; 11.2% reported less than a high school degree. Nearly three-quarters (*n* = 243, 71.5%) reported unemployment in the past 6 months with desire for a job. Most identified intravenous use as their primary route (*n* = 299, 87.9%), DOC-heroin (*n* = 241, 70.7%), ≥ 3 times daily use (*n* = 268, 78.6%), and polysubstance use (*n* = 252, 73.9%). However, 43 respondents (12.6%) reported DOC-fentanyl. Respondents reported 4.4 lifetime opioid overdoses on average (SD = 8.4).

**Table 1:** Characteristics of SSP clients in southern Wisconsin with respect to FTS use

Characteristics	*m* (SD) or %			Unadj. *p* value	Adj. *p* value
	All	FTS use	No FTS use		
	*N* = 341	*N* = 274	*N* = 67		
Site				**0.016**	
Beloit	71 (20.8)	52 (19.0)	19 (28.4)		0.798
Kenosha	80 (23.5)	58 (21.2)	22 (32.8)		**0.009**
Madison	79 (23.2)	66 (24.1)	13 (19.4)		0.844
Milwaukee	111 (32.6)	98 (35.8)	13 (19.4)		Ref
Age	35.7 (8.6)	35.4 (8.1)	36.9 (10.3)	0.600	0.807
Gender				0.677	
Man	202 (59.6)	159 (58.5)	43 (64.2)		Ref
Nonbinary	7 (2.1)	6 (2.2)	1 (1.5)		0.987
Trans	0 (0.0)	0 (0.0)	0 (0.0)		
Women	130 (38.3)	107 (39.3)	23 (34.3)		0.988
Race and ethnicity				**0.009**	
Black or African American, non-Hispanic	24 (7.1)	13 (4.8)	11 (16.4)		**0.006**
Hispanic, any race	21 (6.2)	18 (6.6)	3 (4.5)		0.852
Other or multiple categories, non-Hispanic	32 (9.4)	25 (9.2)	7 (10.4)		0.202
White, non-Hispanic	263 (77.4)	217 (79.5)	46 (68.7)		Ref
Education				0.149	
Less than high school degree	38 (11.2)	30 (11.0)	8 (11.9)		0.891
High school, GED or equivalent	162 (47.6)	124 (45.4)	38 (56.7)		Ref
Some college, trade school or 2y college	129 (37.9)	111 (40.7)	18 (26.9)		0.103
4 years college degree or beyond	11 (3.2)	8 (2.9)	3 (4.5)		0.298
Employment				0.669	
Employed and not job-seeking	41 (12.1)	35 (12.8)	6 (9.0)		0.254
Unemployed and not job-seeking	56 (16.5)	44 (16.1)	12 (17.9)		0.489
Unemployed and job-seeking	243 (71.5)	194 (71.1)	49 (73.1)		Ref
County is majority urban	318 (93.8)	255 (93.4)	63 (95.5)	0.536	0.665
Years of drug use	12.2 (8.2)	11.9 (7.9)	13.0 (9.4)	0.494	0.621
Age at first regular drug use	23.5 (7.2)	23.4 (7.4)	23.9 (6.8)	0.381	–
Current drug of choice				**< 0.001**	
Heroin	241 (70.7)	202 (73.7)	39 (58.2)		Ref
Fentanyl	43 (12.6)	36 (13.1)	7 (10.4)		0.428
Opioid analgesics	10 (2.9)	2 (0.7)	8 (11.9)		**< 0.001**
Cocaine or Crack Cocaine	32 (9.4)	23 (8.4)	9 (13.4)		0.336
Methamphetamine	15 (4.4)	11 (4.0)	4 (6.0)		0.354
Synthetics	0 (0.0)	0 (0.0)	0 (0.0)		
Prescription anxiety drugs	0 (0.0)	0 (0.0)	0 (0.0)		
Prescription amphetamines	0 (0.0)	0 (0.0)	0 (0.0)		
Some other drug	0 (0.0)	0 (0.0)	0 (0.0)		
How often do you try to get drugs with fentanyl?				**0.009**	**0.009**
Every time you get drugs	17 (5.0)	17 (6.2)	0		
Most of the time	49 (14.4)	39 (14.3)	10 (14.9)		
Sometimes	102 (30.0)	86 (31.5)	16 (23.9)		
Rarely	71 (20.9)	60 (22.0)	11 (16.4)		
Never	101 (29.7)	71 (26.0)	30 (44.8)		
Usual route of drug use				**< 0.001**	-
Inject or shoot it into a vein	299 (87.9)	255 (93.4)	44 (65.7)		
Inject or shoot it into a muscle	4 (1.2)	2 (0.7)	2 (3.0)		
Snort it	24 (7.1)	10 (3.7)	14 (20.9)		
Smoke it	12 (3.5)	6 (2.2)	6 (9.0)		
Eat or swallow it	1 (0.3)	0 (0.0)	1 (1.5)		
Insert it rectally	0 (0.0)	0 (0.0)	0 (0.0)		
Use skin popping	0 (0.0)	0 (0.0)	0 (0.0)		
Some other method	0 (0.0)	0 (0.0)	0 (0.0)		
Drug of choice use frequency in past 30 days				0.103	0.650
Less than once per day	18 (5.3)	12 (4.4)	6 (9.0)		
1 time per day	15 (4.4)	10 (3.6)	5 (7.5)		
2 times per day	40 (11.7)	30 (10.9)	10 (14.9)		
3 or more times per day	268 (78.6)	222 (81.0)	46 (68.7)		
Polysubstance use^a^	252 (73.9)	210 (76.6)	42 (62.7)	**0.020**	0.764
Lifetime count of opioid overdose	4.4 (8.4)	4.7 (9.1)	3.3 (4.5)	0.202	0.517
Recent treatments used to stop or reduce use^b^					
Mutual help groups	74 (21.8)	60 (22.0)	14 (20.9)	0.847	0.973
Group program like IOP	73 (21.5)	58 (21.2)	15 (22.4)	0.838	0.702
Individual counseling	154 (45.3)	121 (44.3)	33 (49.3)	0.467	0.320
Medications	190 (55.9)	156 (57.1)	34 (50.7)	0.345	0.669
No treatment	81 (23.8)	66 (24.2)	15 (22.4)	0.758	–
Other	12 (3.5)	11 (4.0)	1 (1.5)	0.313	–
Recent medications to stop or reduce use^b,c^					–
Buprenorphine formulations	122 (64.6)	100 (64.1)	22 (66.7)	0.780	
Extended-release naltrexone	14 (7.4)	13 (8.3)	1 (3.0)	0.291	
Methadone	84 (44.4)	75 (48.1)	9 (27.3)	**0.029**	
Other	10 (5.3)	7 (4.5)	3 (9.1)	0.283	

Bold indicates the results that were < 0.05

Adjusted *p*-values represent results of logistic regression to test the association between FTS use and table characteristics, excluding age at first regular use, usual route of use and recent medications due to collinearity

Missing: Gender—2; Race and Ethnicity—1; Education—1; Employment—1; County—2; Years of drug use—3; Age at first regular drug use—2; Current drug of choice—1; Try to get fentanyl—1; Usual route—1; Overdose count – 4; Recent treatments—1; Recent medications—152

Associations with continuous variables were tested using Mann–Whitney-Wilcoxon tests and associations with categorical variables were tested with chi-square tests

*FTS* fentanyl test strips, *m* mean, *SD* standard deviation, *adj* adjusted, *unadj* unadjusted, *GED* general educational development

^a^Polysubstance use is defined as use of more than one category of drugs excluding alcohol and marijuana

^b^Recent treatments refers to past 3 months

^c^Question was limited to individuals who reported using medications in the past 3 months to stop or reduce their drug use

### Individual-level associations with FTS use

In bivariate analyses, FTS was associated with SSP site, race/ethnicity, drug of choice, route of use, polysubstance use, and methadone use. Respondents who used FTS were more often clients in Milwaukee or Madison relative to Kenosha or Beloit (*p* = 0.016). Rates of FTS use were lower for non-Hispanic Black or African American respondents than non-Hispanic white or Hispanic respondents (*p* = 0.009). Respondents who reported DOC-opioid analgesics were less likely to use FTS than respondents with DOC-heroin, fentanyl, cocaine or crack cocaine, or methamphetamines (*p* < 0.001). FTS use was associated with seeking fentanyl (*p* = 0.009). Intravenous use was associated with FTS relative to snorting, smoking, and swallowing (*p* < 0.001). Polysubstance use (*p* = 0.020) and recent methadone treatment i (*p* = 0.029) were associated with FTS use. In contrast, gender, other recent treatments, frequency of drug use, years of drug use, overdose count, urbanicity, education, and employment status were not associated with FTS use. In multivariable regression, site, race and ethnicity, DOC, and seeking fentanyl were associated with FTS use. Specifically, relative to Milwaukee, respondents at Kenosha site were less likely to report FTS use (*p* = 0.009). Relative to non-Hispanic-white respondents, non-Hispanic Black or African American respondents were less likely to report FTS use (*p* = 0.006). Relative to DOC-heroin, DOC-opioid analgesic was associated with not using FTS (*p* < 0.001). More frequently seeking fentanyl was associated with FTS use (*p* = 0.009).

### Characteristics of FTS use

Respondents who reported FTS use were asked additional questions about FTS. Respondents reported using FTS on average 26.7 (SD = 74.4) times, and using drugs tested by someone else 14.4 (SD = 64.9) times. Over one-third reported using FTS most of the time or more with their DOC. Nearly all respondents had received a prior positive FTS result (95.3%); fewer had received a prior negative result (70.8%). Over 80% reported positive results half the time or more with their DOC. Almost one-third reported test result confusion. In addition, 13.1% reported testing after use. Respondents reported testing a variety of drugs including cocaine (27.0%), crack cocaine (32.1%), fentanyl (36.9%), heroin (92.7%), cannabis (4.7%), methamphetamine (19.0%), opioid analgesics (11.3%), and stimulant medications (1.1%). Among those who reported at least monthly use of heroin (*n* = 235), fentanyl (*n* = 146), opioid analgesics (*n* = 80), methamphetamine (*n* = 90, 32.8%), and cocaine or crack cocaine, 99.1% reported testing heroin, 56.8% reported testing fentanyl, 16.3% reported testing opioid analgesics, 42.2% reported testing methamphetamine and 55.7% reported testing cocaine or crack cocaine, respectively. Notably, 61.3% reported using FTS to test for fentanyl analogs (Table 2).

**Table 2:** Characteristics of FTS use among SSP clients in southern Wisconsin

Characteristics of FTS use	*n* = 274
	*m* (SD) or *n* (%)
How many times have you tested drugs with a FTS?^b^	26.7 (74.4)
How many times have you used drugs tested by someone else with a FTS?^b^	14.4 (64.9)
How often do you test your drugs when using your drug of choice?	
Never^c^	12 (4.4)
Rarely	47 (17.2)
Some of the time	115 (42.0)
Most of the time	64 (23.4)
Every time	36 (13.1)
Has the test strip ever showed there was fentanyl in your drugs? Yes	261 (95.3)
Has the test strip ever showed there was no fentanyl in your drugs? Yes	194 (70.8)
Has the test strip result been confusing so you could not tell if there was fentanyl? Yes	79 (28.8)
How often do you get a positive result when you use a fentanyl test strip on your drug of choice?	
Never	9 (3.3)
Rarely	44 (16.1)
About half the time	88 (32.1)
Most of the time	97 (35.4)
All of the time	36 (13.1)
Do you usually test your drug of choice with a test strip before or after you use? After	36 (13.1)
Which of the following drugs have you tested using a fentanyl test strip?^d^	
Cocaine	74 (27.0)
Crack cocaine	88 (32.1)
Fentanyl	101 (36.9)
Heroin	254 (92.7)
Marijuana	13 (4.7)
Methamphetamine	52 (19.0)
Opioid analgesics	31 (11.3)
Other^e^	7 (2.6)
Stimulant medications like Adderall	3 (1.1)
Have you tried using a fentanyl test strip to look for a fentanyl analog in your drugs? Yes	168 (61.3)

*FTS* fentanyl test strip, *SSP* syringe service program, *m* mean, *SD* standard deviation

^a^The sample includes respondents who reported using drugs tested by themselves or by others with FTS

^b^Three respondents who used drugs tested with FTS skipped these questions

^c^The twelve individuals (4.4%) who reported never using FTS to test their drug of choice were mostly comprised of individuals who had only used drugs tested by someone else (*n* = 10), one individual who used FTS on drugs other than their drug of choice, and one individual who likely answered this question incorrectly (given that they reported testing their drug of choice in a different question)

^d^Respondents were able to select multiple drugs. All respondents who used drugs tested with FTS checked at least one option

^e^Other drugs were pressed Xanax (*n* = 2), MDMA (*n* = 2), pressed pills such as Xanax and Percocet (*n* = 1), urine (*n* = 1), and “speedball,” heroin mixed with cocaine (*n* = 1)

### Situations prompting FTS use

Respondents were asked how often the following situations prompt FTS use: *when you get a new baggie or batch*, *when your supplier puts out a new batch*, *your supplier says the batch is strong*, and *your drugs look different from what you have used before*. For all situations, respondents reported using FTS some of the time to most of the time (Table 3). Specifically, the highest scores were observed when a supplier says the batch is strong (mean: 2.7; SD: 1.2) compared with getting a new baggie or batch (mean: 2.4; SD: 1.1). In analyses not shown, increased respondent age was associated with increased FTS use when the supplier says the batch is strong (*p* = 0.032). DOC-heroin was associated with increased FTS use when the supplier puts out a new batch (*p* = 0.038). Other sociodemographic and drug use characteristics were not associated with situational factors.

**Table 3:** Frequency of FTS use in specific situations among SSP clients in southern Wisconsin who use FTS

Reason	Mean (SD)
You get a new baggie or batch?	2.4 (1.1)
Your supplier puts out a new batch?	2.6 (1.1)
Your supplier says the batch is strong?	2.7 (1.2)
Your drugs look different from what you have used before?	2.5 (1.1)

When you have a fentanyl test strip, how often do you use a test strip to test your drugs if… (0–4: *never, rarely, some of the time, most of the time, every time)*

Key Likert questions were mapped from 0 to 4 and described by the mean (SD)

*FTS* fentanyl test strip, *SSP* syringe service program, *SD* standard deviation

### Logistical factors related to FTS use

Respondents were asked which of the following factors would make getting FTS easier: *having test strips delivered to you, having regular transportation, getting more test strips at a time,* and *getting them at other locations* (in addition to SSP). Respondents reported that all four factors would make it somewhat to quite a bit easier to get FTS (Table 4). Transportation and additional distribution locations exhibited the highest mean score (2.8). In analyses not shown, younger respondents reported increased ease of FTS access with delivery (*p* = 0.038) or regular transportation (*p* = 0.006) relative to older respondents. Respondents with polysubstance use also reported increased ease of FTS access with FTS delivery (*p* = 0.027). Respondents with DOC-heroin (*p* = 0.006), fentanyl (*p* = 0.001), and methamphetamine (*p* = 0.007) reported higher scores for receiving FTS from additional locations relative to respondents reporting DOC-cocaine or crack cocaine. Other sociodemographic and drug use characteristics were not associated with logistical factors impacting access to FTS (Table 5).

**Table 4:** Logistical factors impacting access to FTS among SSP clients in southern Wisconsin who use FTS

Factor	Mean (SD)
You could have them delivered to you?	2.5 (1.1)
You had regular transportation?	2.8 (1.1)
You could get more test strips at a time from Vivent Health?	2.6 (1.2)
You could get them at other locations like the pharmacy or health department?	2.8 (1.2)

How much easier would it be to get test strips if… (0–4: not at all easier, slightly easier, somewhat easier, quite a bit easier, a great deal easier)

Key Likert questions were mapped from 0 to 4 and described by the mean (SD)

*FTS* fentanyl test strip, *SSP* syringe service program, *SD* standard deviation

**Table 5:** Psychosocial factors impacting the likelihood of using FTS among SSP clients in southern Wisconsin

Factor	Mean (SD)
Your counselor or doctor recommended them?	2.0 (1.2)
The SSP staff recommended them?	2.3 (1.2)
Your supplier recommended them?	2.1 (1.4)
Your friends recommended them?	2.2 (1.2)
Someone showed you how to use them?	2.1 (1.3)
You had a safe or private place to use them?	2.3 (1.3)
You didn’t have to waste drugs to use them?	2.7 (1.3)

How much more likely would you be to use fentanyl test strips if… (0–4: not at all likely, a little more likely, somewhat more likely, quite a bit more likely, a great deal more likely)

Key Likert questions were mapped from 0 to 4 and described by the mean (SD)

*FTS* fentanyl test strip, *SSP* syringe service program, *SD* standard deviation

### Psychosocial factors related to FTS use

Respondents were asked how likely the following factors would increase FTS use: *recommendations to use FTS from a counselor or doctor, SSP staff, suppliers, and friends; receiving a demonstration on how to use FTS; having a safe or private place to use FTS; and not needing to waste drugs to use FTS*.

Of the seven factors, not needing to waste drugs received the highest score (mean: 2.7, SD: 1.3). Respondents also identified having a safe or private place (mean: 2.3, SD: 1.3) and receiving recommendations to use FTS from SSP staff (mean: 2.3, SD: 1.2) as factors that would make them somewhat to quite a bit more likely to use FTS. Recommendations from counselor or doctor received the lowest score (mean: 2.0, SD: 1.2).

In analyses not shown, prior FTS use was associated with increased likelihood of FTS use if recommended by a counselor or doctor (*p* = 0.011), by a supplier (*p* = 0.013), or recommended by friends (*p* < 0.001) relative to respondents without prior FTS use. Respondents reporting DOC-methamphetamines reported increased likelihood of FTS use with recommendations from SSP staff (*p* < 0.001), suppliers (*p* < 0.001), and friends (*p* = 0.001) relative to other DOC. Respondents reporting injection use reported increased likelihood of using FTS if recommended by suppliers (*p* = 0.02) or friends (*p* = 0.05) relative to respondents reporting non-injection use. Other individual level characteristics were not significantly associated with psychosocial factors.

## Discussion

In a sample of SSP clients in southern WI, individuals who use FTS more often report DOC-heroin, polysubstance use, injection drug use, and recent methadone treatment compared with individuals who do not use FTS. As such, FTS use might be more common among people with severe addiction and/or at higher risk of overdose [34–36].

We also identified populations not yet using FTS. Individuals who use FTS were less likely to report DOC-opioid analgesics, and rates of testing opioid analgesics were quite low among individuals with regular opioid analgesic use. Rates of stimulant testing were also low among respondents with regular stimulant use, particularly for methamphetamine. Yet, respondents reported a high level of fentanyl penetration consistent with state overdose mortality data [37]. These findings align with recent evidence that a high proportion of co-involved stimulant-opioid overdose occurs among individuals not intending to use opioids [38]. Rising rates of fentanyl adulteration and co-involved stimulant-opioid overdose [38, 39] call for increased educational outreach about the risks of fentanyl in nonopioid substances [39, 40] and wider access to robust drug checking services [41, 42].

We observed disparities in FTS use by race and ethnicity, with non-Hispanic whites more likely to use FTS than non-Hispanic Blacks, Hispanics, or non-Hispanic other or multiple races. These findings reinforce literature documenting racial disparities in receipt of harm reduction services [43–45]. Given the disproportionate growth in opioid-involved overdose among Black and Latinx PWUD [46, 47], it is critical that public health and harm reduction agencies expand efforts to ensure equitable access to FTS supplies and education.

We found that a large set of situational, logistical and psychosocial factors influence FTS use. For example, respondents reported that suppliers’ advisement about a new batch or potency prompt FTS use. Respondents also reported that transportation and additional distribution sites (e.g., pharmacy or health department) impact FTS use alongside mobile delivery services. These findings recommend multiple approaches to reducing geographic barriers. Poignantly, respondents reported that having a safe or private place to use promotes FTS use, which aligns with recent qualitative work [25] and supports the role of overdose prevention sites in reducing overdose risk [48–50]. Finally, SSP staff recommendations may impact FTS use more than recommendations from counselors and doctors, though these findings may reflect limited access to health care [51], as evidenced by low rates of recent treatment or counseling.

Several findings carry safety implications. First, almost two-thirds of FTS users report FTS use some of the time or less. However, FTS results on a single dose cannot assess the entire source. As a result, the ability of FTS to identify fentanyl requires testing the entire drug source or testing with each use. Our findings recommend interventions that support more frequent FTS use.

Second, we found that 13% of respondents who use FTS report testing after drug use. While post-consumption drug checking can inform future use, pre-consumption drug checking has greater potential to impact overdose risk. Thus, intentionally discussing the timing of FTS use could be an important opportunity to increase the impact of FTS on overdose risk.

Third, we found that almost 30% of respondents reported confusion about prior test results, matching other studies [25]. While we did not confirm education receipt with each respondent, the SSP from which we recruited participants provides one-on-one education (in addition to printed materials) from harm reduction specialists about a variety of topics including how to use and interpret FTS. The educational material included in the FTS kit is standard across the SSP locations included in this study. Given this context, persistent confusion about test interpretation calls for additional research with PWUD to identify effective interventions. In addition, 60% reported using FTS to look for fentanyl analogs. However, while FTS can detect a large number of analogs, it is possible for an FTS test to produce false negatives [52, 53]. As such, a negative test result cannot guarantee the absence of analogs. Confusion and misunderstanding when interpreting FTS results could increase overdose risk and indicates a clear role for more robust drug checking services [41, 42] and additional research exploring FTS analog detection in real world samples.

Finally, we found that 70% of respondents report trying to find drugs that contain fentanyl. Given these findings, it is important that providers and educators emphasize harm reduction techniques that acknowledge the likelihood of fentanyl use, such as possessing multiple doses of naloxone (overdose antidote) and not using alone.

Several study findings inform efforts to expand FTS use. First, relative to non-FTS users, FTS users reported a greater likelihood of increasing FTS use with recommendations from health care providers, suppliers, or peers. Thus, these strategies may not be as effective to encourage initial FTS use. Second, we found that concern related to wasting drugs influenced the likelihood of using FTS. Correcting this misperception among PWUD could help expand use of FTS. Third, FTS use was associated with SSP site, with greater FTS use in more urban locations. This variation may reflect differences in transportation barriers, which respondents identified as important factors impacting FTS use. Increasing geographic availability of FTS across diverse locations might help overcome these challenges.

This analysis involved several limitations. First, our sample only included SSP clients. As a result, our findings may reflect the perspective of individuals interested in harm reduction services. Second, our sample is limited to southern Wisconsin, which is more urban than the northern half of the state. Third, to ensure the privacy and protection of respondents, we collected no identifying data. As a result, we could not ensure that respondents did not participate multiple times or in both study phases.

## Conclusions

In sum, we find that SSP clients who use FTS are more often non-Hispanic white, report DOC-heroin and seek fentanyl relative to those who do not use FTS. Frequent positive test results confirm a high level of fentanyl penetration in the southern Wisconsin drug market. While the rate of FTS use to check heroin is high, the rate of FTS use to check illicit stimulants remains low even among individuals with regular stimulant use. Factors that prompt FTS use include supplier reports of a new batch or increased potency and recommendations to use FTS by SSP staff, among others. To expand FTS use, our findings suggest (a) focusing on populations less inclined to use FTS (those reporting DOC-opioid analgesics or a non-opioid, and PWUD who are Black, Hispanic and other nonwhite races and ethnicities); (b) increasing the number and diversity of FTS distribution locations; and (c) correcting misperceptions about wasting drugs when using FTS. Notably, in the course of delivering FTS education, providers should highlight the importance of pre-consumption testing, anticipatory guidance in the setting of confusing results, and limitations of FTS regarding fentanyl analogs. Finally, given the high proportion of PWUD seeking fentanyl, health care providers and harm reduction staff should not assume that FTS will promote fentanyl avoidance. In particular, counseling should emphasize additional behaviors and harm reduction techniques (e.g., naloxone use and not using alone) that PWUD can employ to mitigate risk of overdose when using drugs that test positive for fentanyl.

## Data Availability

The datasets generated and analyzed during the current study are not publicly available at the time of publication.

## Abbreviations

FTS: Fentanyl test strips
PWUD: People who use drugs
SSP: Syringe service program
UWSMPH: University of Wisconsin School of Medicine and Public Health
DOC: Drug of choice
